# Needle‐shaped diatom frustules in food as a possible promoter of esophageal squamous cell carcinoma in coastal southeastern China: A pilot study

**DOI:** 10.1002/ijc.70394

**Published:** 2026-02-23

**Authors:** Haisheng Wu, Yang Li, Lihua Ran, Zhiying Xia, Pi Guo, Shenxi Deng, Erica Conway, Yuan He, Jun Zheng, Huachen Zhu, Linwei Tian

**Affiliations:** ^1^ School of Public Health The University of Hong Kong Hong Kong SAR China; ^2^ Key Laboratory of Marine Ecosystem Dynamics, Second Institute of Oceanography Ministry of Natural Resources Hangzhou Zhejiang China; ^3^ State Key Laboratory of Satellite Ocean Environment Dynamics Second Institute of Oceanography, Ministry of Natural Resources Hangzhou Zhejiang China; ^4^ Department of Preventive Medicine Shantou University Medical College Shantou Guangdong China; ^5^ Heping Hospital Changzhi Medical College Changzhi Shanxi China; ^6^ Institute of Wheat Research, State Key Laboratory of Sustainable Dryland Agriculture Shanxi Agricultural University Linfen Shanxi China; ^7^ State Key Laboratory of Emerging Infectious Diseases The University of Hong Kong Hong Kong SAR China; ^8^ Joint Institute of Virology (Shantou University and The University of Hong Kong), Guangdong‐Hongkong Joint Laboratory of Emerging Infectious Diseases Shantou University Shantou Guangdong China

**Keywords:** diatom frustules, esophageal squamous cell carcinoma, needle‐shaped frustules, non‐mutagenic tumor promoter, trash fish

## Abstract

Emerging genomic evidence suggests that non‐mutagenic promoters shape the global distribution of esophageal squamous cell carcinoma (ESCC). One suspected promoter is a group of minuscule needle‐shaped glass fragments as outgrowths on edible weeds, millet bran, and wheat bracts, which in a bolus of food could scarify a narrowed gullet in high‐risk regions of South Africa, Iran, and northern China. Repeated injuries and chronic inflammation of esophageal epithelium caused by these glass needles are analogous to cosmetic micro‐needling in dermatology‐stimulating collagen and elastin via wound‐healing cascades. While tumors can be unhealed wounds and siliceous “micro‐needles” from wheat bracts have been linked to the high ESCC risk in wheat‐based eaters in semi‐arid northern China, we here report abundant needle‐shaped diatom frustules (siliceous cell walls of diatoms, aka “glass boxes”) in trash fish‐related food samples from high‐ESCC regions of coastal southeastern China. Through a preliminary feeding trial conducted on rats, we found that needle‐shaped diatom frustules may be more likely to lodge in the esophageal epithelium of rodents than round‐shaped ones. Diatom needle contaminants in the diet may underlie the ESCC epidemic in coastal southeastern China, where dietary culture allows the guts of trash fish to enter human food.

## Graphical abstract

What's new?

Esophageal squamous cell carcinoma (ESCC) incidence varies worldwide, suggesting that local environmental and dietary factors are influential. In coastal southeastern China, food products derived from small filter‐feeding fish are important dietary components, though their consumption increased ESCC risk. This study examined the relationship between ESCC and microscopic silica cell walls (frustules) of diatoms that contaminate these fish. Analyses reveal abundant needle‐shaped diatom frustules in common foods derived from these small fish. Feeding trials in rats indicate that these pointed frustules are more likely to lodge in the esophageal epithelium than round frustules, suggesting that they are physical irritants and potentially promote ESCC development.

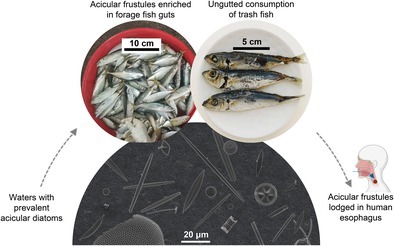

**Abbreviations**
ESCC: esophageal squamous cell carcinoma
H_2_O_2_
: hydrogen peroxide
HCl: hydrochloric acid
MD‐VF‐Auto SEM: microwave digestion‐vacuum filtration‐automated scanning electron microscopy
NSEE: nitrososarcosinethylester
SEM: scanning electron microscope

## 1 INTRODUCTION

The stark geographic contrast in esophageal squamous cell carcinoma (ESCC) incidence around the world implies exogenous factors underlying the etiology.^1^ Despite the fact that specific mutation spectra can be signatures for certain exogenous mutagenic exposures in oncogenesis (e.g., red meat consumption and colorectal cancer), an international genomic study incorporating 552 ESCC genomes from eight countries with varying incidence rates did not pinpoint any mutational signature linked to known or putative risk factors.^2^ Instead, their results showed a remarkable similarity in the mutational signatures between these genomes from high‐ and low‐incidence countries, suggesting it might be some non‐mutagenic promoter mechanisms that set apart the high‐ and low‐risk regions worldwide.^1^

Weed‐ and crop‐derived siliceous spicules in human food have long been suspected as non‐mutagenic ESCC promoters in regions with extremely high ESCC incidence, including South Africa, Iran, and northern China.^3^, ^4^, ^5^, ^6^, ^7^, ^8^ One of these crop‐derived silica needle contaminants has been purified and found to be a more potent cancer promoter than phorbol ester in mice treated with a low dose of cancer initiator.^9^ When silica (glassy) needle contaminants in a bolus of food lodge in a narrowed gullet, they can act on the esophageal epithelium in a way similar to microneedling in dermatology, where microneedling on the skin is employed for cosmetic purposes to initiate skin repair and rejuvenation. In dermatology, the underlying mechanism of microneedling involves minimal skin injuries that damage the epidermis and trigger the cascade of wound healing, skin cell proliferation, and tissue remodeling. Microneedling of the esophageal epithelium, however, could result in acute and chronic esophagitis without any cosmetic benefits. The cases of glass dust esophagitis have exemplified the acute injury caused by dietary intake of minuscule glass fragments by accident.^10^ Worse yet, cancer development could be a larger threat due to repeated injuries and wound healing of the esophagus epithelium, as tumors can be just wounds that do not heal.^11^, ^12^

**FIGURE 1:**
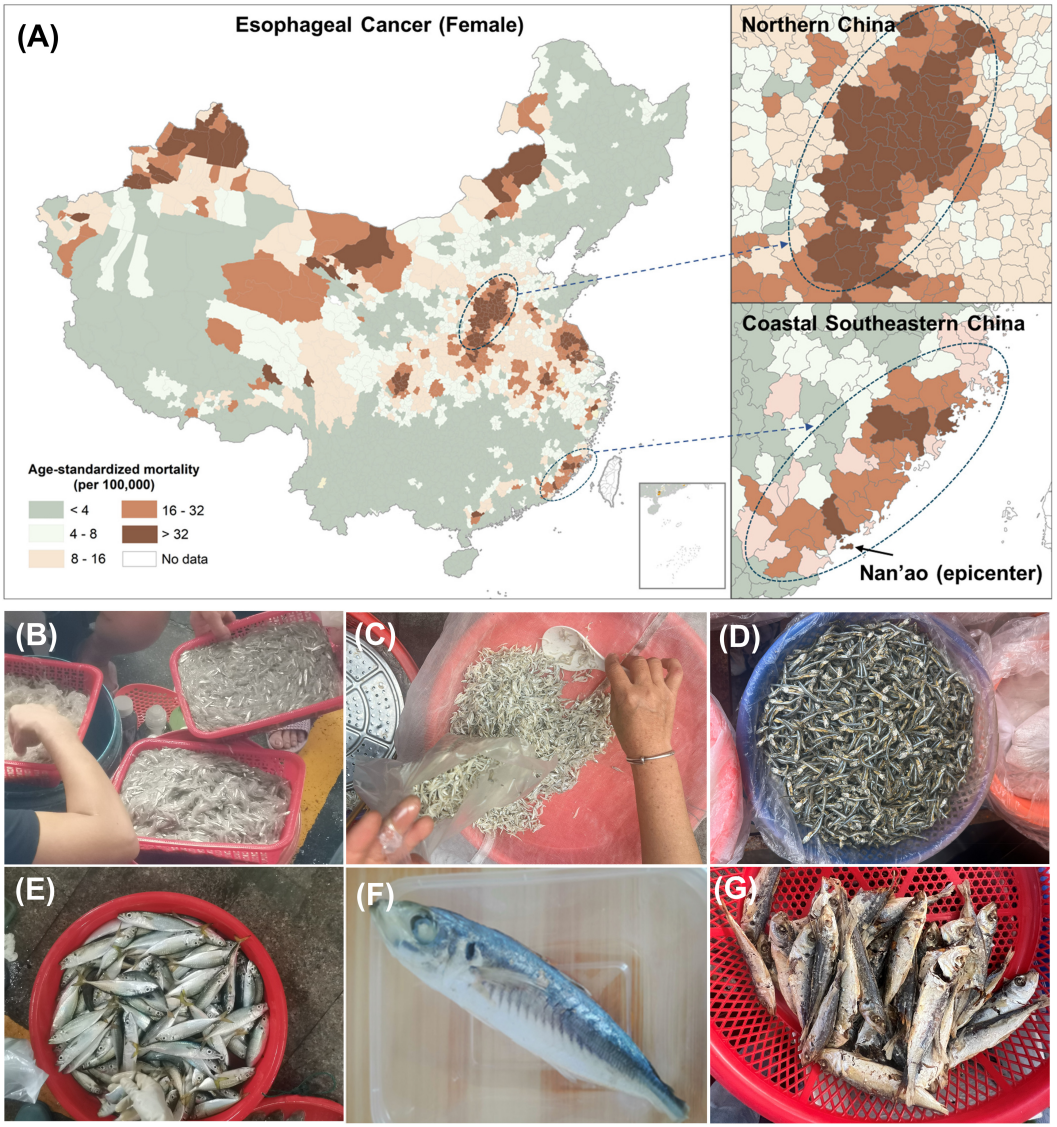
Esophageal cancer risk across China and trash fish‐related dietary culture in high‐risk areas of coastal southern China. (A) The map of female age‐standardized mortality of esophageal cancer in China, based on the only county‐level cancer mortality census data between 1973 and 1975.^16^ The geographic patterns are similar between males (not shown) and females. Dotted circles highlight the esophageal cancer epidemic areas of northern China and coastal southeastern China. No updated national cancer mortality census data at the county level are available as the subsequent surveys were based on stratified sampling methods, but the latest cancer registration indicated no changes in the geographic patterns of esophageal cancer in the past decades.^17^ (B–G) Photos of trash fish taken from the wet markets in Nan'ao Island, the esophageal cancer epicenter of coastal southeastern China. Panels (B) and (C) show raw (fresh) and cooked juvenile anchovies, respectively; Panel (D) shows dried anchovies. The anchovies are usually less than 10 cm in length; panels (E–G) are raw (fresh), steamed, and dried larger trash fish (*Decapterus maruadsi*), respectively. The dried *D. maruadsi* is about 10 cm long, and the steamed one is about 15 cm long. Commonly, the relatively larger *D. maruadsi* is utilized for the preparation of steamed “fish rice,” a local culinary practice where an entire fish is salted, steamed, and consumed in a manner similar to rice, without removing guts.

In northern China, hairy outgrowths on the wheat bracts are siliceous phytoliths (“silica bodies”) in the form of trichomes, which have been suggested to act as a potential non‐mutagenic promoter for ESCC development.^7^, ^8^, ^13^ Aridity and siallite soil (rich in silica) in northern China enhance silica deposits in trichomes on wheat bracts; subsequently, these siliceous spicules can infiltrate and contaminate wheat flour, lodge in the esophagus epithelium, and potentially promote carcinogenesis through repeated injuries and chronic inflammation.^13^ Association between wheat intake and an elevated risk of ESCC was revealed in a case–control study; the combination of high wheat consumption and low water intake increased ESCC risk by more than threefold.^14^ With little consumption of wheat flour, however, coastal southeastern China is another well‐recognized ESCC epidemic region (Figure 1A). With respect to dietary patterns, China generally has a wheat culture in the north and a rice culture in the south. Just like wheat bract, rice husk also has a high silica content and contains phytoliths, but these phytoliths have trapezoidal shapes^15^ and are thin and much lighter than the rice grains. The chance for these rice husk phytoliths to contaminate rice and lodge in the esophagus is low. If not siliceous phytoliths from wheat or rice husks, are there any other dietary suspects for the ESCC epidemic in coastal southeastern China?

In coastal southeastern China where small trash fish‐related dietary culture is popular (Figure 1B–G), fish sauce and dried trash fish have been identified as predominant risk factors for ESCC.^18^ Fish sauce is produced by the hydrolysis of whole trash fish in the presence of salt through natural fermentation over months or years.^19^ Unfiltered homemade fish sauce was shown to have dramatic promoter effects on the carcinogenic process initiated by nitrososarcosinethylester (NSEE) in the forestomach of mice.^20^ Such a promoter effect was not detected in relatively cleaner commercial fish sauce,^20^ which usually undergoes filtration through a filter with a pore size as small as 0.1 μm. What promoter suspect was filtered out here? Could the promoter be another type of glass needles in the guts of trash fish that often contaminate fish sauce and dried fish? As these small trash fish are usually filter‐feeding or low‐trophic‐level, they mainly feed on diatoms, and thus the diatom frustules (highly siliceous cell walls of diatoms, aka “glass boxes”) could be another source of glass needle contaminants.

Therefore, this study aimed to investigate the potential of diatom frustules contaminating the local diet in ESCC endemic areas of coastal southeastern China. We further aimed to characterize the morphology of diatom frustule contaminants and simulate the dietary exposure process.

## 2 METHODS

### 2.1 Collection of trash fish‐related samples

We collected samples from Nan'ao County, the ESCC epicenter of coastal southeastern China (Figure 1A). Nan'ao is an island county, providing a natural research site. With the growth of tourism and economic development on Nan'ao over the past decade, unfiltered homemade fish sauce has become rare in local households, having been largely replaced by more convenient commercial products. Therefore, the presence and characterization of possible diatom frustule contamination were initially investigated by analyzing the residue after filtering the fish sauce, which is known as “fish earth” and looks like diatomaceous earth. Specifically, we randomly collected five samples of fish earth, each weighing 1 kg, from the Nan'ao Fish Sauce Factory. These fish earth samples were subsequently dried and thoroughly mixed, resulting in a total mass of 2.1 kg. Meanwhile, we also collected trash fish‐related food samples, including cooked and dried products of small shrimps, *Stolephorus chinensis* (an anchovy species) and *Decapterus maruadsi* (locally referred to as “Balang”), which are common trash fish‐related dietary components for locals and are typically consumed whole either freshly cooked or dried (Figure 1B–G). Small shrimps and juvenile anchovies are perishable but valued for nutrition, with fresh ones sold in the early morning and replaced by cooked ones to avoid spoilage. Balang is made into a popular dish “fish rice” (a whole fish is steamed by salt water, and eaten like rice without removing any internal organs). Excessive trash fish are made into sun‐dried products, which can be found everywhere in local markets and stores.

### 2.2 Diatom analysis of trash fish‐related samples

For the fish earth and cooked and dried food samples, sample processing and permanent slides preparation for microscopic observations were based on the well‐established method by Dr. Ran^21^: (a) To remove calcareous and organic matter, 10% hydrochloric acid (HCl) and 30% hydrogen peroxide (H_2_O_2_) were added to each sample, and then each sample was heated; (b) after complete digestion, the mixture was centrifuged three times to remove the remaining HCl, H_2_O_2_, and reaction solution; (c) gelatin was added to the remaining mixture to accelerate particle settlement; (d) the remaining sample was carefully decanted into a petri dish containing two already‐fixed 24 mm × 24 mm cover slips, and left to settle for 12–24 h afterward; (e) subsequently, a strip of absorptive paper was used to remove any remaining supernatant in the petri dish; (f) when the material was completely dried, the cover slips were transferred onto a labeled slide mounted with ultraviolet curing adhesive. TM1000 HITACHI, Lecia DM 750A, and Motic BA410 microscopes were used for diatom identification and counting.

### 2.3 Rat feeding experiment and diatom analysis of esophageal tissues

Sixteen 15‐week‐old F344 rats were randomly divided into an exposure group and a control group, with eight rats in each group. The exposure group was administered a diet formulated by mixing the standard feed with fish earth (termed “fish earth diet”). The control group received standard feed alone, with all other environmental and husbandry conditions maintained identically between the two groups. Eight weeks after the initiation of the feeding trial, esophageal tissues were randomly harvested from six rats in the exposure group and three rats in the control group. These tissue samples were subsequently subjected to quantitative diatom analysis. Concurrently, representative samples of the fish earth diet were also collected and analyzed to determine the baseline diatom load and morphology in the exposure source.

The microwave digestion‐vacuum filtration‐automated scanning electron microscopy (MD‐VF‐Auto SEM) method was adopted for processing rat esophageal tissue samples and analyzing diatoms.^22^ Firstly, tissue samples were mixed with 8 mL pure concentrated nitric acid for a 30‐min pre‐digestion process. Subsequently, the samples were transferred to a microwave digestion system (Tianyi Instrument Manufacturing [Chengdu] Co., Ltd., TM‐12a) where a programmed temperature digestion was carried out. Following digestion, a vacuum filtration system (Shenzhen Meinimei Technology Co., Ltd., VF600) was utilized to concentrate the diatom cells from the digestion solution onto the surface of a 0.45 μm pore size polyethersulfone filter membrane. Finally, after undergoing gold plating treatment, the samples were analyzed using a scanning electron microscope (Tescan Trading [Shanghai] Co., Ltd., VEGA3). The automated image acquisition and diatom identification were performed under a magnification of 1000× and a pixel resolution of 1024 × 1024. The results of the automatic diatom detection were further verified by two professionals. To ensure a comprehensive assessment of diatom frustule load, our classification and enumeration protocol incorporated diatom frustule fragments in addition to intact frustules. Fragments were included only if they could be reliably confirmed as originating from either the elongate/needle‐shaped or other diatom morphotypes.

## 3 RESULTS

Abundant diatom frustules were identified in fish earth samples. Each gram of fish earth contained 0.33 million diatom frustules, a figure close to that in diatomaceous earth from sediments of the South China Sea.^23^ Elongated and needle‐shaped diatoms, such as *Thalassionema* spp., *Fragilaria* spp., and *Delphineis* spp., were predominant in the fish earth sample. In the coastal water in the ESCC epidemic area of southeastern China, needle‐shaped *Thalassionema nitzschioides* and *Thalassionema frauenfeldii* have been demonstrated to be the predominant diatom species in the phytoplankton community, accounting for 72% of the total phytoplankton biomass.^24^ The abundance of needle‐like *Thalassionema* spp. in the guts of cooked or dried trash fish is similar to that in fish earth.

**FIGURE 2:**
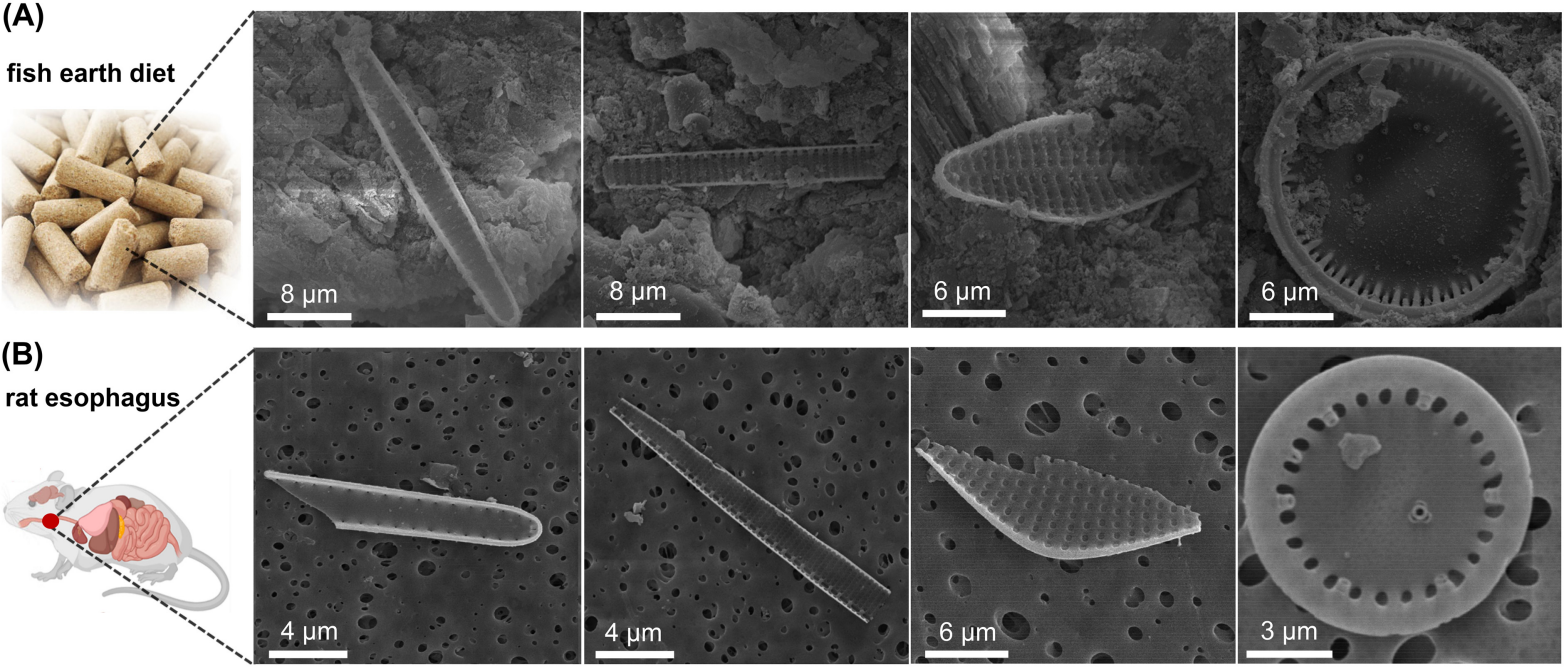
Scanning electron microscopy images of typical diatom frustules in rat feeding experiments. (A) Diatom frustules or their fragments detected from fish earth diet (fish earth added to standard rat feed), left to right: *Thalassionema* spp., *Pseudo‐nitzschia* spp., *Delphineis* spp., and *Cyclotella* spp. (B) Diatom frustules or their fragments detected from the rat esophagus samples, with the same diatom species as in panel (A).

**FIGURE 3:**
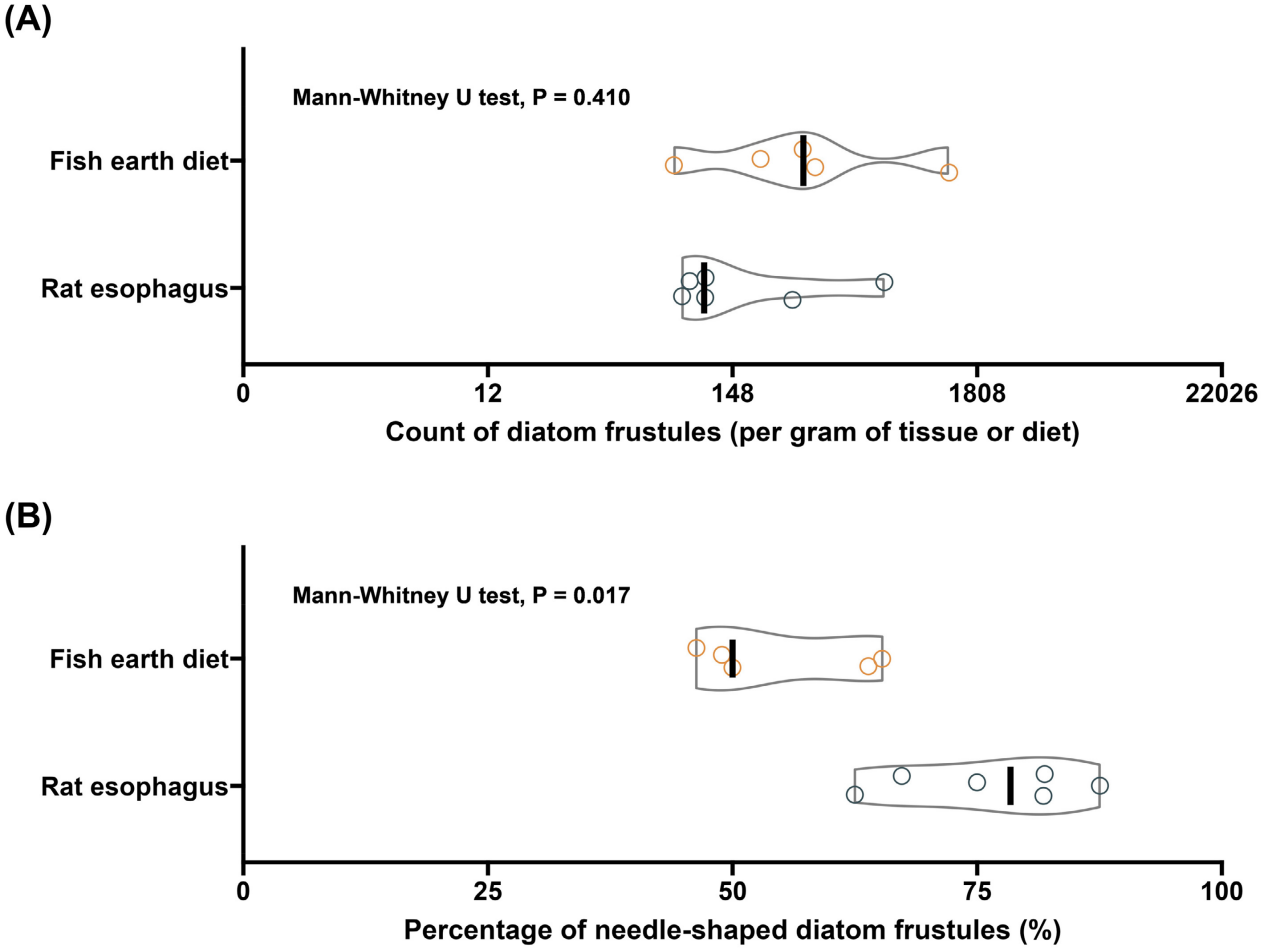
Comparison of the count and composition of diatom frustules in fish earth diet (fish earth added to standard rat feed) and rat esophageal tissue. (A) Violin plots showing diatom frustule counts per gram of fish earth diet and rat esophagus tissue. Individual data points are overlaid on the plots, with black vertical lines representing the median values. It is noted that significant non‐diatom impurities remain in the fish earth diet sample after the microwave digestion process, potentially impacting the accuracy of diatom counting and identification. (B) Violin plots showing the percentage of needle‐shaped diatom frustules in the fish earth diet and the rat esophageal tissue.

A preliminary animal feeding trial was performed to examine the potential of diatom frustules lodging in the rodent esophageal tissue. Results show that the diatom frustule load in the exposure group (fish earth diet) was substantially higher, ranging from 115 to 687 frustules and fragments per gram of rat esophagus, compared to the control group (range: 22–60 per gram). Within the exposure group, the majority were fragments of elongated and needle‐shaped diatom frustules from the fish earth (Figure 2). The percentages of needle‐shaped diatom frustules in rat esophageal tissue were significantly higher than in the fish earth diet (*p* = .017), showing that needle‐shaped diatom frustules were more likely to lodge in esophageal tissue of rodents than round‐shaped ones (Figure 3).

## 4 DISCUSSION

A dietary culture of eating trash fish without removing the guts would be a necessary condition for diatom frustules contributing to any ESCC epidemic in the local populations. Unfortunately, the ESCC epidemic area of coastal southeastern China is particularly well known for its dietary culture of consuming trash fish, which are processed into fish sauce, dried fish, salted fish, and “fish rice.” While most other coastal provinces of China use their trash fish for animal feed, it is only the ESCC epidemic areas of coastal southern China that value trash fish as human food. This geographic contrast in the human consumption of trash fish was disclosed in a report by Greenpeace East Asia which is concerned about the smaller mesh sizes of fishing nets and sustainable fishery.^25^

Not all the coastal waters in China have needle‐shaped diatoms as predominant species in the phytoplankton community; not all the coastal regions in China have the dietary culture of trash fish on the dining table. The unfortunate combination of certain environmental conditions and dietary cultures seems to be restricted to the ESCC epidemic areas of coastal southeastern China. This pattern reminds us again of the possible glass roots of ESCC clusters in China. Currently, we are planning animal experiments to further explore the potential role of diatom needles as a non‐mutagenic promoter in the development of ESCC. Our findings would have food safety implications beyond the ESCC epidemic in China when trash fish is consumed by a much larger population given the modern development in cold storage and transportation services around the globe. Our findings would also warrant greater attention to non‐genotoxic mechanisms of carcinogenesis.

## AUTHOR CONTRIBUTIONS

**Haisheng Wu:** Conceptualization; data curation; investigation; writing – original draft; formal analysis; software; visualization. **Yang Li:** Methodology. **Lihua Ran:** Methodology; resources. **Zhiying Xia:** Methodology; investigation. **Pi Guo:** Methodology; resources. **Shenxi Deng:** Writing – review and editing. **Erica Conway:** Writing – review and editing. **Yuan He:** Resources. **Jun Zheng:** Methodology; resources. **Huachen Zhu:** Writing – review and editing. **Linwei Tian:** Conceptualization; methodology; resources; supervision; project administration; funding acquisition; writing – review and editing; validation.

## FUNDING INFORMATION

This work was supported by the following research grants: the Health and Medical Research Fund (HMRF) from the Food and Health Bureau (FHB) of Hong Kong (Ref. No.: 18192061; 20211551), the General Research Fund (GRF) scheme of the Hong Kong Research Grants Council (Ref. No.: 17613819; 17115122; 17109723), the Theme‐based Research Scheme (TRS) of the Hong Kong Research Grants Council (Ref. No.: T24‐508/22‐N), the General Program of National Natural Science Foundation of China (Ref. No.: 82473595, 82173469), the Guangdong Natural Science Fund (GD‐NSF) of China (Ref. No.: 2022A1515011151), the Seed Funding for Strategic Interdisciplinary Research Scheme from the University Research Committee at the University of Hong Kong (Ref. No.: 102010191), the Non‐profit Central Research Institute Fund of Chinese Academy of Medical Science (Ref. No.: 2020‐PT320‐005), the Chinese Academy of Medical Sciences Innovation Fund for Medical Sciences (Ref. No.: 2021‐I2M‐1‐044), and Theme based Research Scheme (TRS) (Ref No. T24‐508/22‐N).

## CONFLICT OF INTEREST STATEMENT

The authors declare that they have no conflict of interest.

## ETHICS STATEMENT

This research strictly adhered to ethical guidelines and obtained the corresponding ethical approval. The ethical approval for the animal feeding experiment was granted by the Animal Ethics Committee of Changzhi Medical College (no. DW2024038).

## Data Availability

The data that support the findings of this study are available from the corresponding author upon reasonable request.
